# Feasibility and Acceptability of a Mindfulness App Intervention for Healthcare Worker Families Under Stress: A Pilot Micro-Randomized Trial

**DOI:** 10.3390/healthcare14050681

**Published:** 2026-03-07

**Authors:** Sun-Kyung Lee, Sydni A. J. Basha, Qiyue Cai, Abigail H. Gewirtz

**Affiliations:** 1 T. Denny Sanford Harmony Institute, Arizona State University, Tempe, AZ 85287, USA; sunkyung.lee@asu.edu; 2 Reach Institute, Department of Psychology, Arizona State University, Tempe, AZ 85287, USA; sabasha@asu.edu (S.A.J.B.); qiyue.cai@asu.edu (Q.C.); 3 Paul Baerwald School of Social Work, Hebrew University of Jerusalem, Jerusalem 9190501, Israel

**Keywords:** feasibility, acceptability, mindfulness intervention, healthcare workers, wearable technology

## Abstract

**Background:** This pilot study examined the feasibility, acceptability, usability, and preliminary outcomes of *apt.mind*, a mobile app-based mindfulness intervention with an exploratory smartwatch component, among healthcare worker families during the COVID-19 pandemic. **Methods:** Using a micro-randomized trial (MRT) design, 102 healthcare workers and co-parents of children aged 4–13 years were randomized once per day over 30 days to one of three conditions: (1) an audio-guided mindfulness exercise delivered via the *apt.mind* mobile app, (2) an in-app push notification prompting a brief mindfulness activity, or (3) no intervention. Feasibility was assessed through participant enrollment, retention, and daily engagement rates, while acceptability and usability were evaluated through qualitative and quantitative feedback. Exploratory multilevel analyses examined proximal effects of intervention conditions on momentary stress. **Results:** Retention was high, with all participants completing the 30-day protocol, and 80% of participants completed at least one daily survey. Participants reported moderate-to-high acceptability and usability. However, smartwatch battery life and sensor reliability limited the collection of usable physiological data. Multilevel analyses did not identify any significant main effects of intervention condition on momentary stress, but age moderated the association between the audio exercise condition and stress, benefiting older participants. **Conclusions:** Mobile-based mindfulness interventions appear feasible and acceptable for healthcare worker families in high-stress contexts. Although proximal stress effects were limited and exploratory, the findings inform future optimization of just-in-time adaptive interventions. Improvements in wearable technology and MRT implementation strategies are needed to enhance physiological data quality and reduce assessment-related anxiety.

## 1. Introduction

Parental stress adversely affects both caregiving practices and child outcomes [1]. Elevated levels of stress interfere with parents’ ability to regulate their emotions and behaviors, leading to less effective parenting [2]. As a result, children exposed to chronic parental stress are more likely to experience emotional and psychological difficulties [3]. According to family stress theoretical models, external stressors such as economic hardship or significant life disruptions, like the COVID-19 pandemic, can increase parental stress, which in turn negatively impacts child adjustment through pathways such as increased parental mental health problems and disrupted parenting practices [4]. Addressing parental stress is essential, especially among those in demanding professions such as healthcare, where job stress may compound the pressures of parenting.

### 1.1. Healthcare Worker Families During Pandemic

The COVID-19 pandemic placed unprecedented stress on families of healthcare workers globally, not only because of increased professional demands but also due to challenges in their personal lives [5,6]. For healthcare worker families with dependent children, the pandemic disrupted regular routines due to the closure of schools and childcare facilities, exacerbating the already high levels of stress these parents were facing. In addition to longer work hours and heightened personal safety risks, many healthcare workers had to isolate themselves from their families to protect them from potential COVID-19 exposure, further straining their mental health [7,8]. This situation created a dual burden, wherein healthcare workers and their families had to navigate both the physical demands of the pandemic and the emotional toll of being away from their loved ones for extended periods.

Studies have shown that stress among families of healthcare workers during the pandemic reached alarming levels. Research indicates that a significant proportion of healthcare workers experienced symptoms of depression, anxiety, and post-traumatic stress disorder (PTSD) during this period [8]. In fact, pooled prevalence rates estimate that 21–23% of healthcare workers experienced depression, 22–23% experienced anxiety, and nearly 40% struggled with insomnia [9]. These psychological symptoms not only affect healthcare workers’ personal well-being but also compromise their professional functioning, leading to decreased job performance and an increased risk of medical errors [10]. Moreover, these heightened stress levels place children at an increased risk of developing emotional and behavioral issues as a result of exposure to parental stress [3].

### 1.2. Mindfulness-Based Interventions

There is a growing need for effective interventions that can mitigate the stress experienced by healthcare worker parents, particularly during times of crisis like the COVID-19 pandemic. Mindfulness-based interventions refer broadly to the class of interventions promoting key tenets of mindfulness, including present moment awareness, acceptance of feelings and thoughts, and not judging people and situations [11]. Mindfulness has been widely recognized as effective in reducing stress and improving emotional regulation [12]. Mindfulness is taught through strategies such as breathing and meditation and is delivered in programs of varying length and dose for a broad range of populations [13]. In particular, recognizing that extensive training in meditation may be time- and cost-prohibitive for many, brief mindfulness interventions have been developed, which have been shown to be beneficial to psychological health [14]. However, even brief traditional/face-to-face mindfulness programs are often not scalable or accessible for parents who are dealing with high daily demands, especially those in healthcare professions [15,16]. To address these barriers, technology-delivered mindfulness approaches have gained traction, including just-in-time adaptive interventions (JITAIs) and micro-randomized trials (MRTs). JITAIs are designed to deliver support at moments when it is most needed, based on contextual information and real-time data [17]. An MRT, in contrast, is a study design used to optimize such interventions by repeatedly randomizing participants to different intervention options (or no intervention) at multiple decision points over time. This allows researchers to test the proximal effects of intervention components in real-world settings, informing the timing, type, and tailoring of future JITAIs [18].

Wearable technology, such as smartwatches, has the potential to inform JITAIs by tracking physiological markers of stress, such as heart rate variability (HRV), and delivering mindfulness exercises at moments when parents need them most [17,18]. Ecological momentary assessments (EMAs) can further enhance the effectiveness of these technologies by capturing real-time data on perceived stress and emotional well-being in a parent’s natural environment. Studies utilizing these methods have demonstrated their potential to provide interventions that are both highly personalized and ecologically valid [19,20].

Mindfulness interventions themselves are increasingly being incorporated into technology-assisted platforms due to their ability to foster awareness and emotional regulation [21]. By encouraging parents to focus on the present moment without judgment, mindfulness-based approaches can help reduce stress and improve parenting behaviors. This, in turn, benefits their children’s emotional and behavioral outcomes [22,23,24]. When paired with wearable devices that deliver timely, on-the-go support, mindfulness interventions can become especially valuable for parents in high-stress, demanding professions such as healthcare and emergency response. These tools could offer stress relief during unpredictable schedules, helping to prevent stress from escalating into more severe emotional or behavioral issues. However, as far as we are aware, there are no studies examining the feasibility and acceptability of delivering mindfulness interventions through mobile and wearable platforms for parents in high-stress populations like healthcare [25]. Prior studies have demonstrated the potential of such interventions among other populations but are limited by small sample sizes, lack of generalizability, or a focus on other populations [18].

### 1.3. Current Study

The current study addresses a gap in the literature on stress management for parents in demanding healthcare professions and their co-parents, particularly during public health emergencies such as the COVID-19 pandemic. To our knowledge, no prior studies have used MRT design to examine mindfulness-based interventions specifically for healthcare worker parents and their families. This study aims to assess (i) the feasibility, (ii) acceptability, (iii) usability and (iv) preliminary outcomes of a mindfulness intervention delivered through a mobile application paired with a piece of wearable technology. Unlike a fully adaptive JITAI, this pilot MRT used fixed, random daily prompts to establish baseline evidence for timing and format before moving toward a fully tailored JITAI in future work. In this pilot, we focused on self-reported EMAs and survey data, while wearable-derived physiologic signals were treated as exploratory feasibility data.

The intervention combined app-delivered mindfulness exercises with a smartwatch component intended for exploratory real-time stress tracking. Given the pilot nature of this study, we did not use stress tracking to time the delivery of the intervention; rather, the intervention was delivered at a random time each day. Using a micro-randomized trial design, participants were assigned daily to one of three conditions: audio-guided mindfulness activities, a push notification suggesting brief mindfulness activities, or no intervention. Key areas of interest were the participants’ engagement with the study, satisfaction with the technology and activities, responsiveness to EMAs, and the feasibility of wearables to collect physiological data. The study also explored whether the intervention could be effectively integrated into the busy, unpredictable schedules of parents in stressful and highly demanding occupations and whether it led to reductions in stress levels. These findings could set the foundation for future interventions that can better support parental mental health in high-stress environments.

## 2. Methods

### 2.1. Participants

Eligible participants were healthcare workers with regular patient contact and COVID-19 exposure through their job or through their co-parent living with at least one child aged 4–13 years at home. To participate, individuals needed to agree to wear a smartwatch for 30 days, demonstrate English proficiency at a fifth-grade level or higher, and have access to a smartphone throughout the study period. Recruitment was conducted via emails sent to medical and healthcare associations and hospitals in a midwestern state, using professional listservs. Interested individuals accessed a URL to complete the eligibility screening.

**Figure 1:**
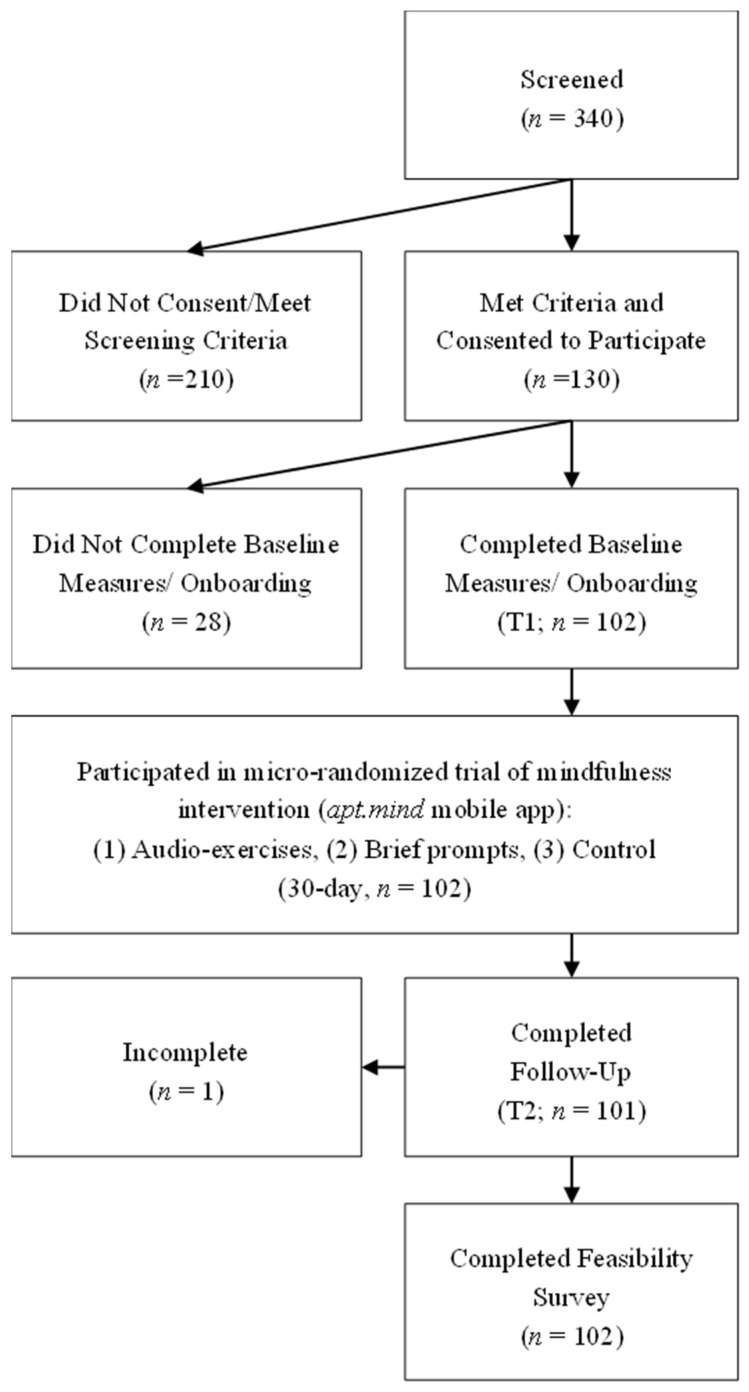
CONSORT diagram. Note. Recruitment period: March 2021 to August 2021. Participants were excluded if they had no children aged 4–13 living with them, did not cohabitate with an eligible co-parent, or worked in healthcare without regular patient contact or COVID-19 exposure.

Of the initial 340 people who were screened, 210 (61.7%) did not meet the eligibility criteria. The remaining 130 (38.2%) participants met the screening criteria and also gave consent to participate in the study. However, 28 participants either did not complete their baseline measures or failed to attend an onboarding session with the study coordinator. As a result, the final sample consisted of 102 participants (78.5%). See Figure 1 for a CONSORT diagram.

On average, participants were 39.3 years old (*SD* = 5.5; range 27 to 53 years). Twenty co-parent dyads (*n* = 40 individuals) participated in the study together. All participants identified as cisgender, 78.4% (*n* = 80) identified as female, and just over 74% of participants worked within the healthcare field. Detailed socio-demographic information is provided in Table A1.

### 2.2. Procedures

The current study was approved by the Institutional Review Board (IRB) at the University of Minnesota. Participant onboarding occurred between March 2021 and August 2021, during a period when the United States was experiencing a rise in the Delta variant, surges in COVID-19 cases across pediatric and adult populations, and elevated hospital admissions [26].

### 2.3. Onboarding

Eligible participants first completed baseline measures via an online Qualtrics survey and were subsequently mailed a Fossil Gen 4 Sport Smartwatch (Fossil Group, Richardson, TX, USA). The smartwatch was programmed to collect photoplethysmography (PPG) for an exploration of HRV feasibility. Once they had received the smartwatch, participants then took part in an individual onboarding session conducted via Zoom with the project coordinator. During this session, participants confirmed the receipt of the smartwatch, its charger, an instruction booklet, information about the *apt.mind* app (*apt.mind*; University of Minnesota, USA) including a private username and password, and a $50 visa gift card as compensation for participation. The project coordinator then gathered key information, including wake and sleep schedules on weekdays and weekends, typical work schedules, the feasibility of wearing the watch during work hours, smartphone accessibility at work, and a preferred two-hour daily window to complete the EMAs. Participants were instructed to wear the smartwatch throughout the day, including during work hours when feasible. Participants were also asked about the reliability of their home Wi-Fi, their smartphone type, wrist preference for wearing the watch, and whether the watch fit comfortably. During the onboarding session, participants were also guided through the downloading process of the *apt.mind* app, connecting their watch to their smartphone, and how to use and maintain both devices.

### 2.4. Daily Intervention and Assessments

Throughout the 30-day study period, participants were randomized once per day at midnight to one of three intervention conditions: (1) an audio-guided mindfulness activity delivered via the *apt.mind* mobile app, developed by the research team, offering 2–5 min practices such as inward-focused activities (e.g., sitting, observing, and body scanning), outward-focused activities (e.g., mindful eating and bell ringing), and self-care practices (e.g., kindness and self-compassion); (2) an in-app push notification prompting a brief mindfulness activity (e.g., ‘Hum a song that makes you smile,’ and ‘Think of someone you are grateful for’); or (3) no mindfulness activity or notification (control). The participants were randomized daily with equal probability (1/3 per condition). This study refers to the intervention conditions assigned to the audio-guided activities as ‘audio exercises’ and to the push notifications suggesting brief activities as ‘brief prompts.’

During each day, participants were prompted to complete two sets of EMAs. The first assessment (EMA1) was delivered within a designated 10 min window and assessed current stress. Regardless of whether EMA1 was completed, participants received the intervention or control notification corresponding to their daily randomized condition after the EMA1 response window closed. If EMA1 was completed within the response window, the notification was delivered immediately following submission. If EMA1 was not completed within five minutes, participants received a reminder notification. An hour after the intervention or control prompt, the participants were prompted to complete a second assessment (EMA2), which assessed their current emotions, stress levels, stress regulation strategies, and engagement with the assigned activity. EMA2 had a 60 min window for completion, with a reminder notification if it was not submitted within 30 min. All prompts occurred within a two-hour window, and EMAs were only accessible through the notification system. The participants could earn up to $100 for consistently completing the daily EMA questions ($1.67 per EMA, totaling $3.33 per day for completing both sets).

In addition to the daily prompts, the participants had the option to complete additional mindfulness activities through *apt.mind* whenever they chose, selecting from a variety of exercises suited to their needs at any given time.

### 2.5. Exit Session

After the 30-day study period, participants completed the exit process independently via an online Qualtrics survey sent by the program coordinator. The exit survey covered the same domains as the baseline survey. The participants also participated in an approximately hour-long virtual exit session with the study coordinator, during which they completed a feasibility and acceptability survey and provided feedback on their overall experience in the study. Finally, participants were asked to return their smartwatch by mail and were compensated with an additional $50 for completing the exit process. In total, participants could earn up to $200 for study participation ($50 onboarding + max $100 EMA + $50 exit).

### 2.6. Measures

**Feasibility.** To assess the feasibility of *apt.mind* and a wearable component, this study evaluated its participants’ willingness to engage with and remain in the study, as well as their response rates to the EMAs over the 30-day period. The response rate was measured separately for EMA1 (a 4-item survey with a 10 min response window) and EMA2 (a 15-item survey with a one-hour response window), with any completed response counted as engagement. Based on a prior smart EMA review [27] reporting an average completion rate of 71.6%, a priori feasibility benchmark of ≥70% average EMA completion was specified. Engagement was calculated based on the total number of days participants completed both EMA1 and EMA2. Because brief prompts were delivered as in-app notifications without required app interaction, objective engagement metrics were available only for audio exercises. Audio intervention dose was quantified using app usage logs, including the number of sessions initiated and total duration of audio engagement (in minutes) across the 30-day period. We also assessed wearable feasibility by examining the availability of PPG data that was collected before EMA1 and after EMA2, as well as the total hours of PPG data recorded over a 24 h period. To estimate the participants’ adherence to wearing the device, timestamped PPG data were segmented into daily blocks and rounded to the nearest minute to calculate total wear time and the participants’ daily percentage of use. These estimates served as a proxy for actual device wear.

**Acceptability.** To assess the acceptability of the wearable technology and the *apt.mind* app as a stress intervention tool, participants were asked about their overall satisfaction with using both the smartwatch and the app. Items included questions about the physical comfortability of wearing the watch daily (1 = not at all to 4 = very comfortable), enjoyment using the app for stress management (0 = mostly no to 1 = mostly yes), perceived effectiveness of the app’s audio exercises and the brief prompts for reducing the participants’ stress (1 = not at all to 4 = very much), and whether the notifications and daily EMA prompts were manageable (1 = strongly disagree to 4 = strongly agree). The participants also rated how much they liked wearing the watch (1 = not at all to 4 = very much). In addition, participants were given open-ended questions to provide feedback on their experience with the wearables for stress monitoring, survey reminders, daily EMA questions, and suggestions to improve the overall experience.

**Usability.** Usability was evaluated by examining the participants’ ease of use and ability to engage with both the *apt.mind* app and the smartwatch. Items explored the participants’ ability to follow instructions for the intervention and engage with the technology regularly. The questions included whether other smartwatch features (e.g., calls, texts, and emails) were used (1 = yes and 0 = no), how often they forgot to wear or charge the watch (1 = never to 5 = always), ease of navigating the app (1 = very easy to 5 = very difficult), and ease of doing both the audio exercises and brief prompts (1 = very easy to 5 = very difficult). The open-ended questions included whether participants used the device for other purposes, such as fitness tracking or notifications, and whether they encountered any technological or functional issues with the *apt.mind* app (e.g., app crashing, buttons malfunctioning, pages failing to load, or audio exercises not playing). The participants were also asked to report any issues with the login portal, the progress feature, and the receipt or display of notifications. They were instructed to specify when, how frequently, and in which sections of the app these problems occurred. The participants were encouraged to share any additional insights and suggestions about their experience using the smartwatch and app.

**Psychosocial and Demographic Measures.** The participants completed a series of psychosocial and behavioral assessments at both the onboarding and exit sessions to evaluate their mental health, stress levels, and family functioning.

**Depression.** The Patient Health Questionnaire-9 [28] (PHQ-9) measured parents’ depressive mood (9-item, e.g., “Little interest or pleasure in doing things”) in the past two weeks on a 5-point Likert scale (1 = Not at all to 5 = Nearly every day). Higher sum scores indicated greater levels of depression. Internal consistency was strong at both baseline (α = 0.86) and post-test (α = 0.86).

**Anxiety.** Parents’ anxiety was measured with the PROMIS Anxiety Short Form [29] (v1.0). Participants rated 8 items related to anxious moods (e.g., “I feel nervous”) in the past 7 days on a 5-point Likert scale (1 = *Never* to 5 = *Always*). The T-scores were calculated, with higher scores indicating higher levels of anxiety. The measure demonstrated good reliability at both baseline (α = 0.90) and post-test (α = 0.91).

**Sleep disturbance.** The PROMIS Sleep Disturbance Short Form [29] (v1.0) assessed sleep disturbances with 8 items related to their sleep (e.g., “I had difficulty falling asleep”) in the past 7 days on a 5-point Likert scale (1 = *Very Poor* to 5 = *Very Good*). The T-scores were calculated, with higher scores indicating greater sleep disturbances. The reliability was high at both baseline (α = 0.91) and post-test (α = 0.91).

**Post-traumatic stress.** The Impact of Events Scale-6 [30] (IES-6) was utilized to rate post-traumatic stress symptoms (6-item, e.g., “I thought about it when I didn’t mean to”) in the past 7 days on a 4-point Likert scale (0 = *Not at all* to 3 = *Nearly Every day*). Sum scores across items were calculated, with higher scores indicating greater levels of post-traumatic stress symptoms. The scale showed strong internal consistency at both baseline (α = 0.87) and post-test (α = 0.86).

**Experimental avoidance.** The Acceptance and Action Questionnaire [31] (AAQ-II version 2; 7-item) was used to assess experiential avoidance and psychological inflexibility (e.g., “I’m afraid of my feelings”) on a 7-point Likert scale (1 = *Never True* to 7 = *Always True*). Sum scores across items were calculated with higher scores indicating greater levels of psychological inflexibility. The current sample showed good internal consistency at baseline (α = 0.92) and post-test (α = 0.73).

**Family stress.** The participants reported their COVID-related stress and experiences over the last few weeks by using the COVID-19 Family Stress Scale [32] (FSS; 10-item). Each item was on a 5-point Likert scale (1 = *Strongly Disagree* to 5 = *Strongly Agree*) and the confirmatory factor analysis (CFA) supported three factors, including family issues (e.g., “Loss of or limited childcare”), living issues (e.g., “Housing or utilities”), and personal stress (e.g., “Increased anxiety or depression”). Average scores across items were calculated, with higher scores indicating greater levels of family stress. The measure demonstrated good reliability at baseline (α = 0.88) and post-test (α = 0.89).

**Parental self-efficacy.** The participants rated four items drawn from the Parental Locus of Control—Short Form [33] (PLOC-SF) about parental efficacy (e.g., “I am often able to predict my child’s behavior in situations”) on a 5-point Likert scale (1 = *strongly agree* to 5 = *strongly disagree*). Sum scores across items were calculated with higher scores indicating greater levels of parental efficacy. Internal consistency was moderate at both baseline (α = 0.67) and post-test (α = 0.60).

**Demographics.** The participants’ age, sex, ethno-racial status, and income were gathered. Sex assigned at birth (1 = female and 0 = male) and gender identity were assessed; all participants reported concordant responses. Their race and ethnicity were asked, and we coded those as non-Hispanic, White, and others (1 = White and 0 = non-white). Pre-tax combined annual household income had an ordinal value (from 1 = less than $25K to 8 = $200K or more).

**Heart Rate Variability.** Smartwatches were programmed to collect raw PPG signals at 100Hz for an exploratory assessment of HRV, which reflects beat-to-beat variation in heart rate. HRV reflects the balance between the sympathetic (“fight or flight”) and parasympathetic (“rest and digest”) nervous systems. A higher HRV is generally associated with better adaptability to stress, while a lower HRV is associated with increased stress or a reduced recovery capacity, making it a key indicator of autonomic function and well-being. To examine potential changes in physiological stress surrounding the intervention, the HRV assessment required continuous 5–10 min segments of PPG data collected before EMA1 and after EMA2.

### 2.7. Data Analyses

In this study, a content analysis was conducted to systematically examine the participants’ experiences with the smartwatch, the app, and the survey/reminder features. Content analysis allowed for the quantification and statistical analysis of large volumes of text-based data, facilitating the identification of patterns across participants’ open-ended responses [34]. Qualitative data was drawn from feedback provided during the exit survey. A set of priori codes was developed based on predefined categories related to the participants’ experiences with the smartwatch, the app, the daily surveys, and the reminders. Three authors (SL, SB, and QC) collaboratively coded 15% of the responses to develop a codebook, inductively creating additional codes based on the content of the responses [35] and ensuring consistency among coders. Each coder then independently coded the remaining responses, followed by group discussions to resolve discrepancies and verify accuracy. The final dataset was consolidated across all coders for analysis.

Descriptive analyses were conducted on the quantitative data collected at both the baseline and the exit session, including the EMA response rates over the 30-day study period. Bivariate correlations were used to explore the relationships between the participants’ EMA engagement and their demographic characteristics. To assess changes in psychosocial and behavioral outcomes before and after the intervention, a paired sample *t*-test was performed using SPSS version 26, applying bootstrapping with 1000 resamples.

To examine proximal effects of the intervention on momentary stress, multilevel analysis was conducted using the *lme4* package in R. Given the nested data structure, repeated EMA observations (Level 1) were modeled within individuals (Level 2). The model included time-invariant predictors (age, sex, race/ethnicity, income, and person–mean stress across the study period) and time-varying predictors (study day, intervention condition, prior stress exposure, and prior-day deviations from an individual’s average stress level). Interaction terms between intervention conditions and age and sex were examined to explore potential moderation and to inform future tailoring of interventions. Missing data were handled with maximum likelihood estimation.

## 3. Results

### 3.1. Feasibility

All 102 participants who completed the recruitment and onboarding processes were retained throughout the entire 30-day study period, with only one participant missing the follow-up survey due to an administrative error. However, all participants completed the feasibility survey at the end of the study.

**Figure 2:**
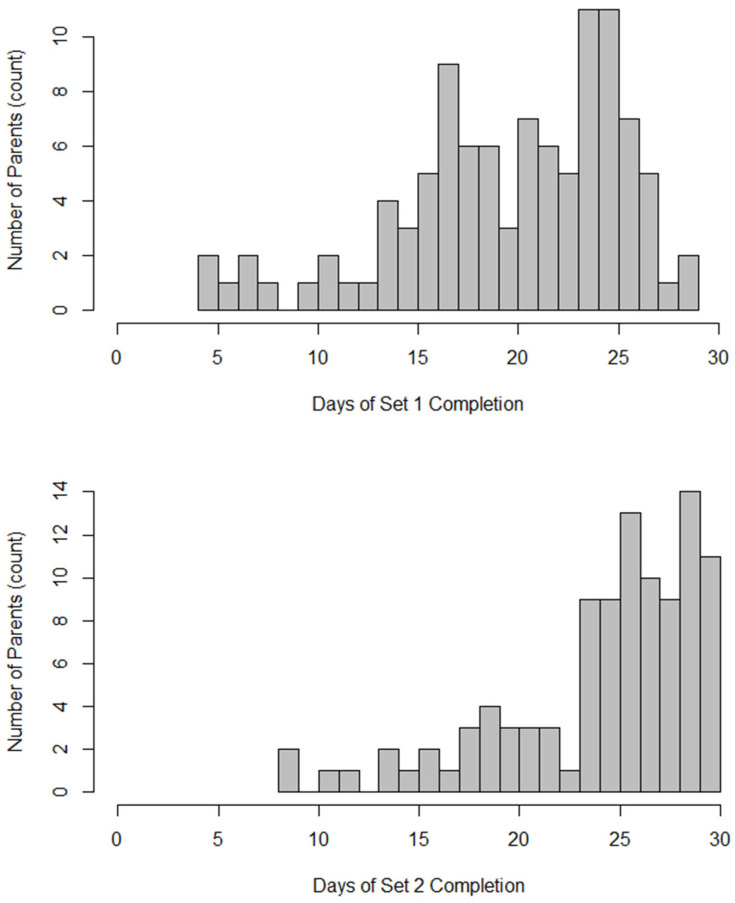
Frequency of EMA survey responses for set one (**top**) and set two (**bottom**).

The response rates for EMA1 and EMA2 surveys provide additional insights into engagement. The participants completed the EMA1 survey on 20.06 days (66.86%; *SD* = 5.62; and range: 4–29), while the EMA2 survey was completed on 24.61 days (82.02%; *SD* = 5.06; and range: 8–30) out of 30 possible days. Thus, the mean completion rate across the two daily EMA prompts was 74.44%. Completion frequency differed significantly between EMA1 and EMA2 (*t* = 14.2 and *p* < 0.001), with EMA2 showing higher adherence. Overall, 60.4% of the participants completed both EMA1 and EMA2 on all 30 study days. The distribution of the survey response frequencies is shown in Figure 2. A strong positive correlation was found between the EMA1 and EMA2 completion rates (*r* = 0.822 and *p* < 0.01). To examine temporal patterns in EMA1 responding, prompts were coded using a 24 h clock (Table A2). The majority of prompts (88%) were delivered between 8:00 and 18:59. The response rates during these daytime hours were relatively stable, generally ranging from 0.63 to 0.72. The completion rates were highest during the early evening period (18:00 to 20:59). Among demographic variables, age was the only demographic characteristic significantly associated with response frequencies. Older age was associated with lower completion rates for both EMA1 (*r* = −0.382 and *p* < 0.01) and EMA2 (*r* = −0.261 and *p* < 0.01).

On average, participants completed almost seven sessions over the 30-day period (range = 0–48 and median = 5 sessions), spending approximately 2.28 min per session. The total engagement time averaged approximately 19 min across the study period (range = 0–138 min and median = 11.43 min).

The overall data showed limited availability of PPG data across participants, with substantial variability in the amount of time the PPG data was available (Table 1). Excluding three participants who experienced sensor issues and had no PPG data collected over the 30 days (*n* = 99), the percentage of PPG sensor data available across the 30-day study period (720 h total) ranged from 0.48% to 56.92%, with a median of 10.98%. This indicates that for nearly half of the participants, PPG sensor data was available for less than 11% of the study period. In terms of actual hours of available PPG data, most participants had fewer than four hours of data on average (*M* = 3.38 h, *SD* = 2.58, and range = 0.12–13.67 h), with a median of 2.6 h. When examining any PPG data availability around EMA prompts, PPG values were available, on average, 68% of the time before EMA1 (21.3 days) with a median of 71% (21.3 days) and 46% of the time after EMA2 (13.8 days) with a median of 44% (13.2 days). However, only about 32% of PPG data (9.6 days) were consistently available both before EMA1 and after EMA2 across the 30-day period, with eight participants having no PPG data available at either time point. These findings suggest challenges in maintaining device adherence, particularly around the key hours of interest, indicating that improvements are needed to enhance data fidelity in future studies.

### 3.2. Acceptability

Qualitative content analysis identified both positive and negative categories within the themes related to the acceptability of the intervention: use of the watch, use of the app, and the survey/reminders (Table 2).

The feedback about the smartwatch was mixed. Participants described the watch as either comfortable and wearable (22.5%), bulky and uncomfortable (18.6%) or bulky (8.8%), with a small subset reporting that wearing the device was anxiety-inducing (5.9%) or not feasible in their work settings (4.9%).

Three themes emerged related to the acceptability of the app: brief prompts, audio exercises, and general activity feedback. Brief prompts were generally viewed positively, with participants describing them as effective for stress management (20.6%) and easy to engage with (6.9%). On the other hand, feedback on the audio exercises was mixed. Some participants found the audio exercises effective (11.8%), whereas others reported concerns about audio voice (10.8%), length (5.9%), or inconvenience (5.9%).

Participants also reported varied reactions to the activities overall. While some described them as effective (11.8%) and enjoyable (6.9%), concerns included limited variety (8.8%) and unclear instructions (2.9%). Regarding the acceptability of the EMA questions, the participants generally found them straightforward (4.9%), although some described the questions as redundant (3.9%).

The quantitative findings supported moderate acceptability (see Table A3). The participants reported a moderate satisfaction with the smartwatch (*M* = 2.65, *SD* = 0.85; 58% “moderately” or “very” satisfied) and a high daily comfort (*M* = 3.16, *SD* = 0.83; 78% “moderately” or “very” comfortable). Notably, 74% of the participants enjoyed using the app for stress management and reported that notifications were reasonable (*M* = 3.50, *SD* = 0.61; 96% “agree” or “strongly agree”), easy to read (*M* = 3.72, *SD* = 0.50; 98% “agree” or “strongly agree”) and easy to understand (*M* = 3.78, *SD* = 0.41; 100% “agree” or “strongly agree”). A paired sample *t*-test indicated that brief prompts were rated as more effective than audio exercises (*t* = 2.82 and *p* = 0.006; Cohen’s *d* = 0.28), including when the participants were with children (*t* = 2.25 and *p* = 0.027; Cohen’s *d* = 0.23) and when they were outside the home (*t* = 2.99 and *p* = 0.004; Cohen’s *d* = 0.30). There were no subgroup differences by age, sex, or healthcare worker status.

### 3.3. Usability

The study also evaluated the usability of both the smartwatch and the app-based intervention (Table 3) from the qualitative and quantitative feedback to understand participants’ interactions with the technology and the challenges they encountered.

Smartwatch usability was mixed. The participants reported both positive experiences (20.6%) and technical challenges (13.7%). The most frequently cited issues involved battery life (84.3%) and PPG sensor reliability (74.5%), with some noting slow performance (11.8%).

App usability feedback reflected three themes: context, connectivity, and app navigation. In terms of context, some participants reported difficulty engaging in activities at work (22.5%) or around children (8.8%), while a few found that the integration into daily routines was manageable (2.9%). Connectivity challenges were noted when Wi-Fi was unavailable (13.7%). App navigation feedback was mixed among those who described the app as user-friendly (20.6%), while others reported issues of keeping the screen awake during audio exercise (15.7%).

The EMA usability concerns were primarily related to survey timing. Many participants reported that the 10 min EMA1 window was too short (40.2%), particularly during work hours. To note, 9.8% indicated that anticipating survey prompts or missing them contributed to feelings of stress or anxiety.

Participants also provided quantitative feedback (see Table A4). Most participants engaged with smartwatch features (60%) and reported rarely forgetting to wear (*M* = 2.25, *SD* = 0.85; 65% “never” or “rarely”) or charge the device (*M* = 2.07, *SD* = 0.94; 69% “never” or “rarely”). App navigation was generally rated as easy (*M* = 1.26, *SD* = 0.63; 94% “somewhat easy” or “very easy”) for both brief prompts (88%) and audio exercises (91%). Older participants reported finding brief prompts easier to use than younger participants (*F* = 4.23 and *p* = 0.04), with no differences observed in sex or healthcare worker status.

### 3.4. Preliminary Efficacy of the Interventions

The bootstrapped paired *t*-test assessed changes in various psychosocial and behavioral outcomes from the baseline (T1) to the post-test/exit session (T2) (Table 3). Most measures showed no significant changes, except for family stress. COVID-19 family stress scores decreased from the baseline (*M* = 2.67 and *SD* = 1.02) to the post-test (*M* = 2.49 and *SD* = 1.05), demonstrating a statistically significant reduction (*t* = −2.28, *p* = 0.019, Cohen’s *d* = 0.23, 95% CI [0.03, −0.42]).

In exploratory multilevel analyses of EMA data (Table 4), experiencing stress in between EMAs was strongly associated with higher momentary stress (*B* = 0.946 and *p* < 0.001). No significant main effects were observed for either brief prompts or audio exercises. However, the interaction between the audio exercise using the *apt.mind* app intervention and age was statistically significant (*B* = −0.02, *p* = 0.04, and 95% CI [−0.04, −0.00]), indicating a small tendency for the audio exercises to be relatively more effective at reducing momentary stress among older participants. No significant age moderation was observed for the brief prompts condition. Additionally, no significant differences in intervention efficacy were observed in the participants’ sex status.

### 3.5. HRV

The study was unable to analyze HRV due to the limited availability of usable PPG data. As described in the feasibility and usability findings, intermittent sensor connectivity and device-related issues substantially constrained the collection of continuous PPG data, particularly during the targeted 5–10 min windows before EMA1 and after EMA2. As a result, the consistency of PPG data was insufficient to support meaningful HRV computation or pre–post comparisons of physiological stress across the study period.

## 4. Discussion

This study explored the feasibility, acceptability, usability, and preliminary outcomes of *apt.mind*, an app-based intervention with an exploratory smartwatch component, to provide momentary stress reduction strategies over a 30-day period. The results offer valuable insights into the participants’ engagement with the technology and their experiences, highlighting both strengths and challenges in the implementation of such an intervention. Overall, the study demonstrated high participant retention, with all 102 participants completing the 30-day study period. The average EMA completion exceeded the a priori feasibility of 70%, consistent with prior EMA research [27]. However, response rates were higher for EMA2 compared to EMA1, and participant feedback indicated that the shorter EMA1 response window was more challenging to complete. Examination of time-of-day patterns showed that EMA1 completion rates were lowest during early morning hours (06:00–07:00; 54–59%) and highest during evening hours (18:00–20:00; 69–72%). This supports the notion that the 10 min response window was less feasible during busy work hours. Older participants showed lower response rates to EMAs.

The limited availability of PPG data, with usable data available for only 10.98% of the study period on average, highlights a key challenge in collecting continuous physiological data over 24 h. Because of insufficient data availability, wearable-derived physiological data could not be incorporated into analytic models and are interpreted as feasibility outcomes using timestamps. This finding underscores the need for improved wearable technology, including battery life and sensor reliability, to enhance data fidelity in future trials. Also, future studies should collect baseline PPG data before the intervention to check the data quality and sensor reliability.

Participants had diverse usability experiences, with the majority of technical challenges stemming from the smartwatch, particularly battery life and PPG sensor reliability. Connectivity challenges also disrupted app-to-watch notifications when Wi-Fi was unavailable. Although the app interface was generally perceived as easy to navigate, the short EMA1 response window and unpredictable notification timings were frequently described as anxiety-inducing. This anxiety reflects features of the assessment design (e.g., response windows and incentive structure). Future research should consider design modifications such as flexible response windows, normalizing occasional missed prompts, and shifting toward activity-focused rather than assessment engagement models.

In this single-group pre–post comparison, a significant reduction in COVID-19-related family stress was observed, while no other psychosocial or behavioral outcomes showed statistically significant changes over the study period. Because this analysis did not compare outcomes across randomized conditions, differences among the intervention groups cannot be inferred. The exploratory multilevel analyses leveraged the MRT design to examine proximal effects on momentary stress. No significant main effects of intervention conditions were observed. It is also important to acknowledge that data collection occurred during the Delta wave of the COVID-19 pandemic. Elevated and sustained stress exposure during the pandemic may have attenuated the short-term effects of mindfulness practices. Moderation analyses indicated the association between the audio exercise and momentary stress varied by age, with older participants showing a relative reduction compared to younger participants. However, these models represent exploratory tests of proximal effects within the MRT framework and should be interpreted cautiously.

These findings inform an optimization roadmap for future JITAI trials collecting physiological data through sensors. First, technological refinement is needed. Battery limitations and sensor instability suggest the need for more energy-efficient sensing algorithms for continuous HRV monitoring. Also, Wi-Fi dependence and notification disruption highlight the importance of offline capability and reliable cross-device synchronization. Second, the EMA timing and response windows should be customizable by users to better align with occupational demands and reduce assessment-related anxiety. Third, the MRT studies can be leveraged to build a more tailored JITAI, incorporating context-aware delivery based on stress events, time-of-day patterns, and user characteristics such as age.

This study is not without limitations. A primary limitation was the inability to analyze HRV data due to low availability of usable PPG sensor data, largely attributable to connectivity instability and battery limitations. This limitation prevented the study from evaluating the physiological impact of the intervention. Although other commercial devices (e.g., Apple Watch, Garmin, and Whoop) may offer more stable sensors, accessing raw physiological data from these platforms typically requires proprietary agreements and restricted backend access. For this study, we selected a programmable device (i.e., a Fossil watch) to enable direct access to raw PPG signals and reduce reliance on proprietary algorithms via the open-source Google Wear OS.

Another limitation is the study population. Our participants included both healthcare workers and their co-parents, and they may experience stress differently, particularly during pandemics. Healthcare workers were exposed to elevated workplace demands and infection risk, whereas co-parents may have experienced secondary stressors related to caregiving, role shifts, or household strain. The study was not powered to formally compare stress processes between these subgroups, and differential stress exposure may have influenced both engagement and intervention responsiveness. Future research should examine occupational role as a potential moderator of both feasibility and efficacy. Similar interventions could benefit individuals in other high-stress occupations, such as other emergency responders (e.g., firefighters and the police), child welfare workers, and military personnel. Replication in more diverse occupational groups and demographic populations will be essential to understanding how such interventions may function across different contexts.

Finally, future studies could incorporate multivariable engagement models, such as generalized estimating equations or mixed-effects models, to identify predictors of adherence and responsiveness. Variables such as age, work shift type, device compatibility, and occupational role may meaningfully predict engagement patterns and intervention effects, allowing for more precise tailoring of future JITAIs.

More broadly, although real-time physiological data have strong theoretical potential to support personalized stress interventions, technological and implementation barriers remain substantial. Addressing these barriers is needed to translate mobile mindfulness interventions into scalable, data-informed tools that can support stress management in high-demand professions.

## 5. Conclusions

In conclusion, this study contributes important feasibility evidence for mobile-based mindfulness interventions that incorporate an exploratory wearable component for stress management in high-stress environments like healthcare. The mobile-based intervention demonstrated moderate acceptability and usability among healthcare worker families. Despite technological challenges, the intervention showed potential in reducing COVID-19-related family stress, and age emerged as a potential tailoring feature for guided mindfulness exercises.

This study also informs the future optimization of digital mindfulness interventions. Future trials should aim to achieve ≥70% EMA completion and collect baseline PPG data to ensure wearable data availability (e.g., at least a continuous 5 min PPG segment) and adjust for individual HRV differences. Refinement of the EMA timing and response windows is recommended, including incorporating user-customizable scheduling options to reduce assessment-related anxiety and improve adherence and data quality.

Generalizability is limited by the relative homogeneity of the sample in gender identity and by the inclusion of both healthcare workers and non-healthcare co-parents who may experience stress differently. Future studies should recruit more demographically and occupationally diverse samples to better understand differential engagement and intervention responsiveness.

As wearable technology advances and implementation strategies are refined, mobile mindfulness interventions integrated within adaptive trial designs may become increasingly effective and scalable tools for supporting stress management and well-being in high-demand professions.

## Figures and Tables

**Table 1:** Descriptive statistics for PPG availability and data collection over 30 days.

	Mean	Min	25% Quartile	50% (Median)	75% Quartile	Max
Total PPG Availability (%)	14.49%	0.48%	6.71%	10.98%	18.42%	56.92%
Total PPG Availability (hours)	3.48	0.12	1.61	2.64	4.43	13.67
Days PPG Available —before EMA1 and after EMA2	9.75 (32%)	0.00 (0%)	4.35 (14.5%)	8.70 (29%)	14.25 (47.5%)	30.00 (100%)
Days PPG Available —before EMA1	20.53 (68%)	0.00 (0%)	15.90 (53%)	21.30 (71%)	25.20 (84%)	30.00 (100%)
Days PPG Available —after EMA2	13.80 (46%)	0.00 (0%)	8.10 (27%)	13.20 (44%)	18.75 (62.5%)	30.00 (100%)

**Table 2:** Themes and categories from qualitative data: acceptability and usability feedback on the watch, the app, and the survey/reminders.

	Category	Theme	Positive	Negative
**Acceptability**	Watch	Watch Style	Watch size/weight (7) Fashionable (8) Durable (1)	Watch was too bulky (9) Not fashionable (1)
		Wearability	Comfortable (23) Can wear at work (1)	Uncomfortable (19) Cannot wear at work (5) Does not like watch (4)
		Study	Watch reminded them to participate in the study (1)	Wearing the watch for the study was anxiety-inducing (6)
	App	Brief prompts	Brief prompts were easier (7) Brief prompts were effective (21)	Did not receive many brief prompts (2) Did not like brief prompts (2) Brief prompts were not effective (2)
		Audio exercises	Liked audio voices (6) Audio was effective (12) Audio was easier (1) Enjoyed guided breathing activity (4) Liked audio exercises (1)	Did not like audio voices (11) Audio was not effective (3) Audio was inconvenient (3) Audio exercises were too long (6) Did not receive many audio prompts (8) Did not enjoy imagination activity (1) Did not like audio activities (1)
		General Activities	Activities were effective (12) Good variety of activities (3) Activities were enjoyable (7) Activities were of good length (2)	Activities diminished in effectiveness over time (1) Not enough variety of activities (9) Activities were uninteresting (1) Activities were not suited to impatient people (1) Activities were anxiety-inducing (3) Activity instructions were unclear (3) Some exercises were better than others (1)
	Survey/ Reminders	Questions	Liked the questions (7) Questions were simple (5) Emotion ratings increased self-awareness (2)	Questions were ambiguous (2) Questions were redundant (4) Questions did not fit the context (2) Questions were uninteresting (1)
**Usability**	Watch	Technology	Functionality (2) Convenient PPG activation (1)	Poor functionality (7) PPG sensor (76) PPG side button (1) Watch was slow/had a touch delay (12)
		Battery/ Charging	Charged quickly (2)	Poor battery life (86) General charging difficulties (7) Other features drain battery (5) Unable to keep charged while at work (3) Wanted a wall adapter (1)
		Use	User friendly (8) Liked receiving notifications (5) Watch features (8) Preferred this watch over others (1) Liked the watch (1)	Not user friendly (9) Did not like receiving notifications on watch (4) Could not receive notifications on watch (1)
	App	Context	Easy to incorporate into life (3)	Could not do when around children/others (9) Could not do when at work (23) Activities did not fit the context (6)
		Connectivity		Does not work when not connected to Wi-Fi (14) Watch and app disconnected (3)
		App Navigation	User friendly (21) Enjoyed the app (3)	Not user friendly (1) App would freeze/crash (4) The progress feature was unclear (3) Did not use the app unless prompted (2) Notification/prompt issues (8) Audio could not keep the screen awake (16)
	Survey/ Reminders	Process	Easy and straightforward (11)	Difficult survey access (4) Issues with survey loading (4) Issues with confirmation/submission (4)
		Length of Survey	Good length of surveys (4)	The second survey was too long (2)
		Timeframe	Timeframe within the window (1) The second survey was timed well (2)	The 10 min window is too short (41) Timeframe was anxiety-inducing (1) Poorly timed (1) Short windows between two surveys (1) Waiting for surveys was anxiety-inducing (5) Missing a survey was stressful (5) Cannot complete survey when at work (7)

Values in parentheses indicate frequency of coded mentions.

**Table 3:** Paired sample *t*-test results for psychosocial and behavioral outcomes.

	Time	Mean	*SD*	*N*	*t*	*p*-Value	Cohen’s *d*	95% CI (Lower, Upper)
Depression	T1	4.61	4.46	101	0.06	0.743	≈0.01	−0.20, 0.19
	T2	4.63	4.40					
Anxiety	T1	56.18	6.64	101	−1.16	0.353	0.12	−0.08, 0.31
	T2	55.53	7.24					
Sleep Disturbance	T1	51.88	8.12	101	−1.43	0.231	0.14	−0.06, 0.34
	T2	51.04	7.92					
Post-traumatic stress	T1	1.12	0.84	101	−0.61	0.498	0.06	−0.13, 0.26
	T2	1.07	0.81					
Experiential Avoidance	T1	17.89	8.34	101	−1.67	0.122	0.17	−0.03, 0.36
	T2	16.80	7.39					
COVID-19 Family Stress	T1	2.67	1.02	101	−2.28	0.019	0.23	0.03, 0.42
	T2	2.49	1.05					
Parental Efficacy	T1	4.49	0.55	98	−1.56	0.136	0.10	−0.10, 0.30
	T2	4.41	0.59					

**Note.** T1 = baseline, T2 = post-test; *SD* = standard deviation, Cohen’s *d* = effect size, CI = confidence interval for effect size.

**Table 4:** Multilevel model predicting momentary stress.

	*B*	SE	95% CI	*t*	*p*
Intercept	−0.249	0.111	[−0.467, −0.032]	−2.248	0.025
Age (mean-centered)	0.001	0.006	[−0.011, 0.013]	0.208	0.835
Female	0.012	0.047	[−0.079, 0.103]	0.256	0.798
White	0.007	0.057	[−0.105, 0.119]	0.123	0.902
Income	0.003	0.012	[−0.020, 0.026]	0.252	0.801
Day in Study	0.002	0.002	[−0.003, 0.006]	0.748	0.454
Stressor Experienced	0.946	0.041	[0.865, 1.026]	23.147	<0.001
Average Stress	0.892	0.032	[0.830, 0.954]	28.279	<0.001
Prior Day Stress Variability	0.004	0.02	[−0.035, 0.042]	0.182	0.856
Intervention (brief Prompts)	0.033	0.046	[−0.057, 0.124]	0.728	0.467
Intervention (*apt.mind* audio app)	0.008	0.046	[−0.083, 0.099]	0.17	0.865
Brief Prompts x age	−0.004	0.009	[−0.021, 0.012]	−0.518	0.604
*apt.mind* audio app x age	−0.018	0.009	[−0.035, −0.001]	−2.064	0.039

**Note.** SE = standard error; CI = confidence interval; and reference = control group.

## Data Availability

The data that support the findings of this study are available from the corresponding author upon reasonable request. The data is not publicly available due to confidentiality restrictions and IRB limitations.

## Appendix A

**Table A1:** Sociodemographic variables for parents by sex.

Demographic	Total (*n* = 102)	Females (*n* = 80)	Males (*n* = 22)
Age (in years): *M* (*SD*)	39.3 (5.6)	39.1 (5.5)	39.9 (5.7)
Parent status: % (*n*)			
Biological parent	83.3% (*n* = 85)	85% (*n* = 68)	77.3% (*n* = 17)
Non-biological parent	4.9% (*n* = 5)	2.5% (*n* = 2)	13.6% (*n* = 3)
Both	5.9% (*n* = 6)	7.5% (*n* = 6)	0% (*n* = 0)
Not provided	5.9% (*n* = 6)	5% (*n* = 4)	9.1% (*n* = 2)
Co-parent in the study: % (*n*)	39.2% (*n* = 40)	25% (*n* = 20)	90.9% (*n* = 20)
Employment status: % (*n*)			
Full-time	89.2% (*n* = 91)	88.8% (*n* = 71)	90.9% (*n* = 20)
Part-time	7.8% (*n* = 8)	8.8% (*n* = 7)	4.5% (*n* = 1)
Homemaker	2.9% (*n* = 3)	2.5% (*n* = 2)	4.5% (*n* = 1)
Employment industry: % (*n*)			
Healthcare worker	74.5% (*n* = 76)	88.8% (*n* = 71)	22.7% (*n* = 5)
Non-healthcare worker	21.6% (*n* = 22)	7.5% (*n* = 6)	72.3% (*n* = 16)
Not provided	2.9% (*n* = 3)	3.8% (*n* = 3)	0% (*n* = 0)
Race: % (*n*)			
White/Caucasian	87.3% (*n* = 89)	87.5% (*n* = 70)	86.4% (*n* = 19)
Hispanic/Latin	2.9% (*n* = 3)	3.8% (*n* = 3)	0% (*n* = 0)
Black/African American	3.9% (*n* = 4)	2.5% (*n* = 2)	9.1% (*n* = 2)
Asian	2.9% (*n* = 3)	3.8% (*n* = 3)	0% (*n* = 0)
Multiracial	3.9% (*n* = 4)	5% (*n* = 4)	0% (*n* = 0)
Not provided	2% (*n* = 2)	1.3% (*n* = 1)	4.5% (*n* = 1)
Relationship status: % (*n*)			
Married	76.5% (*n* = 78)	73.8% (*n* = 59)	86.4% (*n* = 19)
Single/never married	14.7% (*n* = 15)	16.3% (*n* = 13)	9.1% (*n* = 2)
Divorced	6.9% (*n* = 7)	7.5% (*n* = 6)	4.5% (*n* = 1)
Separated	2% (*n* = 2)	2.5% (*n* = 2)	0% (*n* = 0)
Annual household income: % (*n*)			
$25,000–$49,999	8.8% (*n* = 9)	8.8% (*n* = 7)	9.1% (*n* = 2)
$50,000–$74,999	9.8% (*n* = 10)	10% (*n* = 8)	9.1% (*n* = 2)
$75,000–$99,999	17.6% (*n* = 18)	17.5% (*n* = 14)	18.2% (*n* = 4)
$100,000–149,999	22.5% (*n* = 23)	22.5% (*n* = 18)	22.7% (*n* = 5)
$150,000–$199,999	17.6% (*n* = 18)	18.8% (*n* = 15)	13.6% (*n* = 3)
$200,000+	18.6% (*n* = 19)	18.8% (*n* = 15)	18.2% (*n* = 4)
Not provided	4.9% (*n* = 5)	3.8% (*n* = 3)	9.1% (*n* = 2)
Education: % (*n*)			
High school/GED	7.8% (*n* = 8)	2.5% (*n* = 2)	27.3% (*n* = 6)
Associate’s degree	22.5% (*n* = 23)	26.3% (*n* = 21)	9.1% (*n* = 2)
Bachelor’s degree	32.4% (*n* = 33)	31.3% (*n* = 25)	36.4% (*n* = 8)
Master’s degree	27.5% (*n* = 28)	31.3% (*n* = 25)	13.7% (*n* = 3)
Doctoral degree	9.8% (*n* = 10)	8.8% (*n* = 7)	13.7% (*n* = 3)
Licensed in their field: % (*n*)	84.3% (*n* = 86)	92.5% (*n* = 74)	54.5% (*n* = 12)

**Table A2:** Distribution of EMA1 response rates by hour of day.

Hour	Completed	Missed	Total Delivered	Response Rate
0	0	1	1	0.00
1	0	0	0	-
2	1	0	1	1.00
3	1	0	1	1.00
4	2	1	3	0.67
5	2	1	3	0.67
6	7	6	13	0.54
7	50	35	85	0.59
8	117	68	185	0.63
9	156	73	229	0.68
10	188	106	294	0.64
11	192	92	284	0.68
12	185	105	290	0.64
13	150	85	235	0.64
14	122	64	186	0.66
15	151	68	219	0.69
16	136	79	215	0.63
17	175	87	262	0.67
18	171	67	238	0.72
19	112	50	162	0.69
20	33	15	48	0.69
21	16	4	20	0.80
22	5	1	6	0.83
23	4	2	6	0.67

**Note.** Hour coded using a 24 h clock (0 = 00:00 to 00:59; 23 = 23:00 to 23:59).

**Table A3:** Descriptive analysis of the acceptability of the smartwatch, the app, the mindfulness exercises, and the notifications.

Acceptability Questions	N	Mean	SD	Range
**Watch/App**				
How much do you like wearing this watch?	102	2.65	0.85	1–4
How comfortable was it to wear the watch daily?	102	3.16	0.83	1–4
Did you enjoy using an app for stress management?	102	0.74	0.44	0–1
**Audio exercises**				
How effective were the audio exercises for managing stress?	102	2.85	0.68	1–4
Do audio exercises help manage stress when with children?	99	2.49	0.97	1–4
Do audio exercises help manage stress when with adults?	102	2.88	0.88	1–4
Do audio exercises help manage stress when alone?	102	3.40	0.74	1–4
Do audio exercises help manage stress when at home?	102	3.27	0.76	1–4
Do audio exercises help manage stress when at work?	100	2.62	1.01	1–4
Do audio exercises help manage stress when outside?	98	2.77	0.96	1–4
**Brief prompts**				
How effective were brief stress reduction activities?	100	3.09	0.57	1–4
Do brief prompts help manage stress when with children?	101	2.74	0.91	1–4
Do brief prompts help manage stress when with adults?	101	2.91	0.80	1–4
Do brief prompts help manage stress when alone?	101	3.49	0.69	1–4
Do brief prompts help manage stress when at home?	101	3.32	0.71	1–4
Do brief prompts help manage stress when at work?	100	2.69	0.94	1–4
Do brief prompts help manage stress when outside?	101	3.05	0.75	1–4
**Notifications/Questions**				
The number of notifications per day was reasonable.	102	3.50	0.61	1–4
The notifications were easy to read on my mobile device.	102	3.72	0.50	1–4
The notifications were easy to understand.	102	3.78	0.41	1–4
The number of daily questions was manageable.	102	3.49	0.61	1–4

**Table A4:** Descriptive analysis of the usability of smartwatch and app features.

Usability Questions	N	Mean	SD	Range
Did you use the calls, texts, emails, and/or calendar features on the watch?	102	0.60	0.49	0–1
How often, if ever, did you forget to wear your watch?	101	2.25	0.85	1–5
How often, if ever, did you forget to charge the watch?	102	2.07	0.94	1–5
How easy was it for you to navigate the app?	102	1.26	0.63	1–5
How easy or difficult was it to do the audio exercises within the app?	102	1.49	0.90	1–5
How easy or difficult was it to do the brief prompts?	100	1.41	0.78	1–5
